# Supplementary material for: Structural Reorganization of Imidazolium Ionic Liquids Induced by Pressure-Enhanced Ionic Liquid—Polyethylene Oxide Interactions
Source: Int J Mol Sci. 2021 Jan 19;22(2):981. doi: 10.3390/ijms22020981 (PMC7835789; doi:10.3390/ijms22020981)
Supplement: Supplementary file 1 [file ijms-22-00981-s001.pdf]

Supplementary Materials

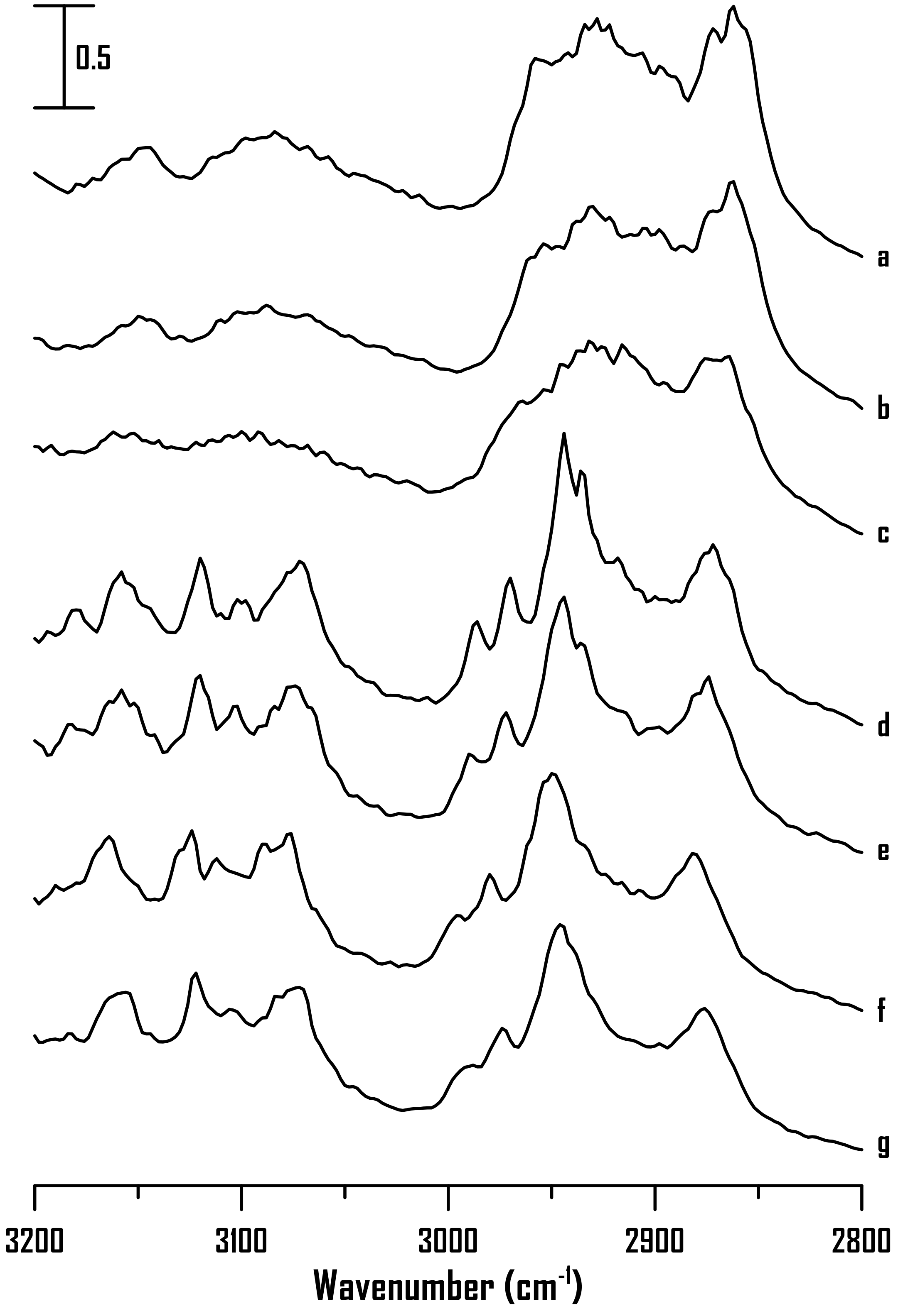

Figure S1. IR spectra of the [HMIM]Br-PEO mixture containing 75 wt% [HMIM]Br at (a) ambient pressure and (b) 0.4, (c) 0.7, (d) 1.1, (e) 1.5, (f) 1.8, and (g) 2.5 GPa.

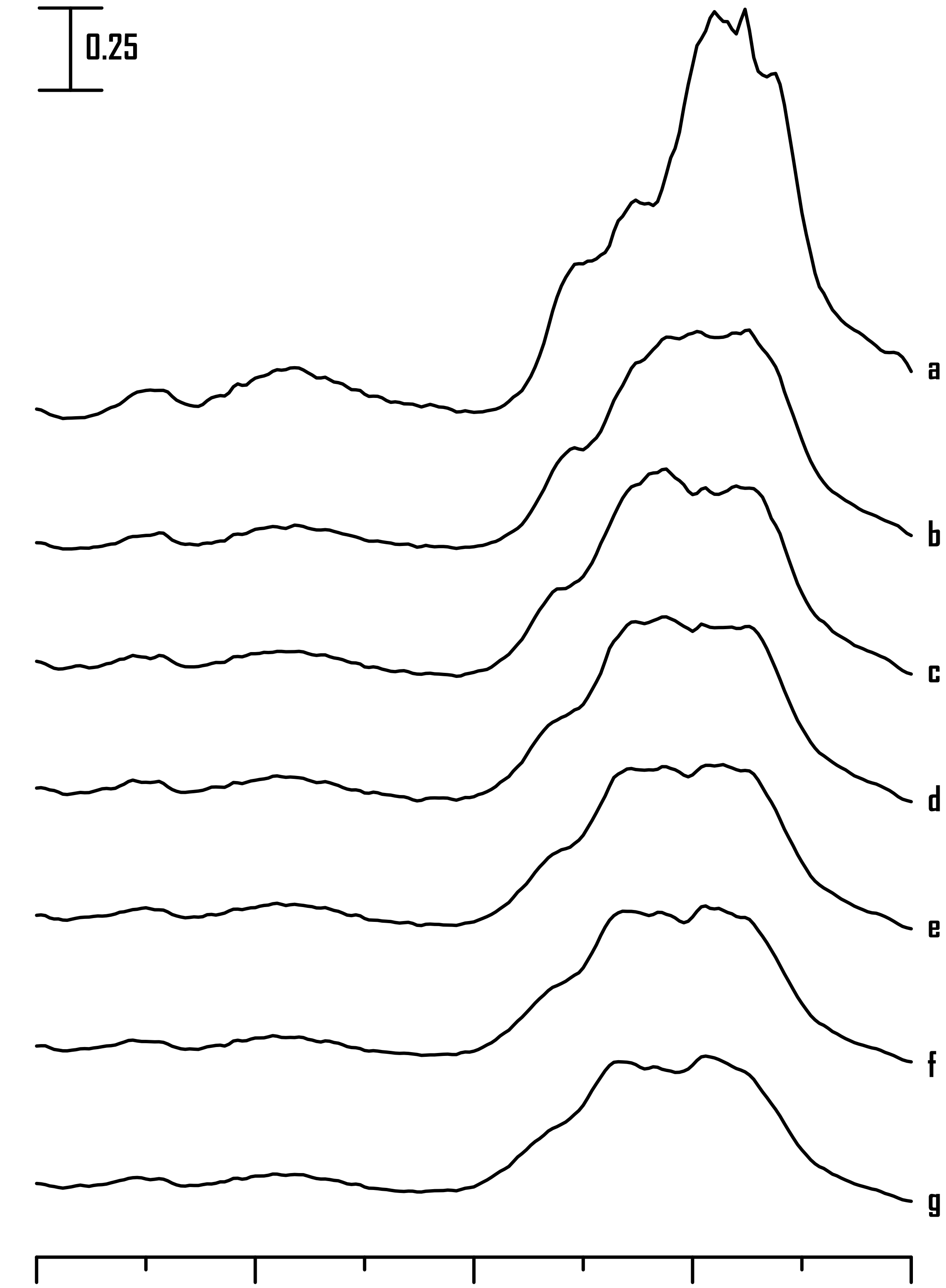

Figure S2. IR spectra of the [HMIM]Br-PEO mixture containing 50 wt% [HMIM]Br at (a) ambient pressure and (b) 0.4, (c) 0.7, (d) 1.1, (e) 1.5, (f) 1.8, and (g) 2.5 GPa.

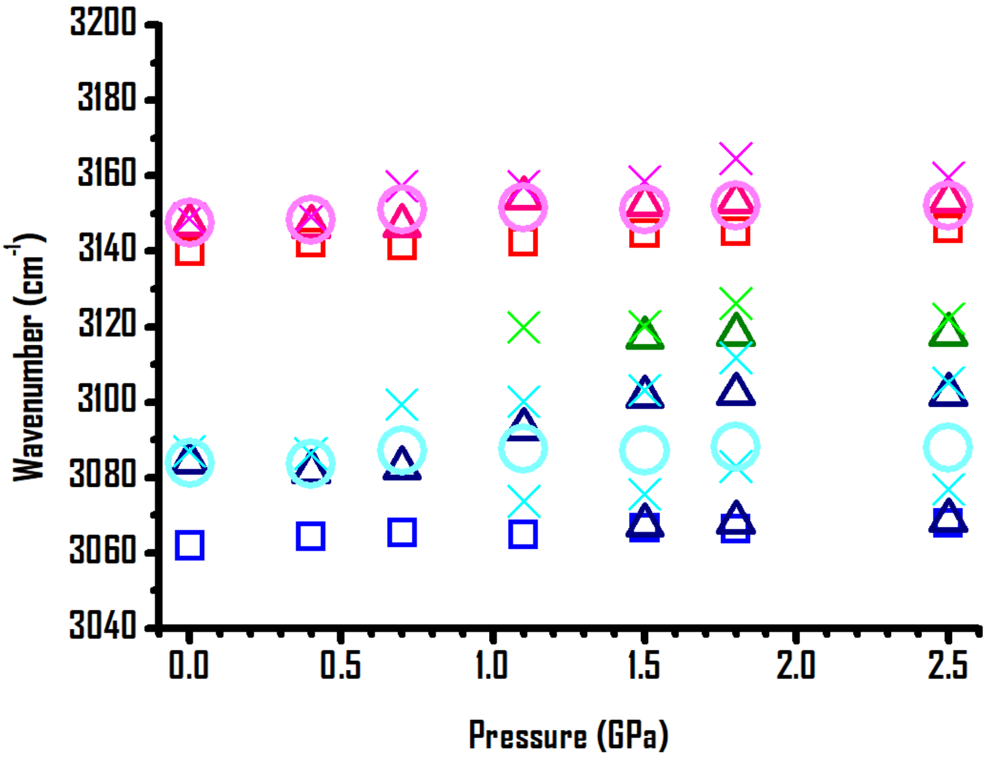

Figure S3. Pressure dependence of the C–H stretching frequencies of imidazolium $C^{4,5}$–H and C^{2}–H of pure [HMIM]Br (squares) and [HMIM]Br-PEO mixtures with 80 wt% (triangles), 75 wt% (cross (X)), and 50 wt% (circle) [HMIM]Br.

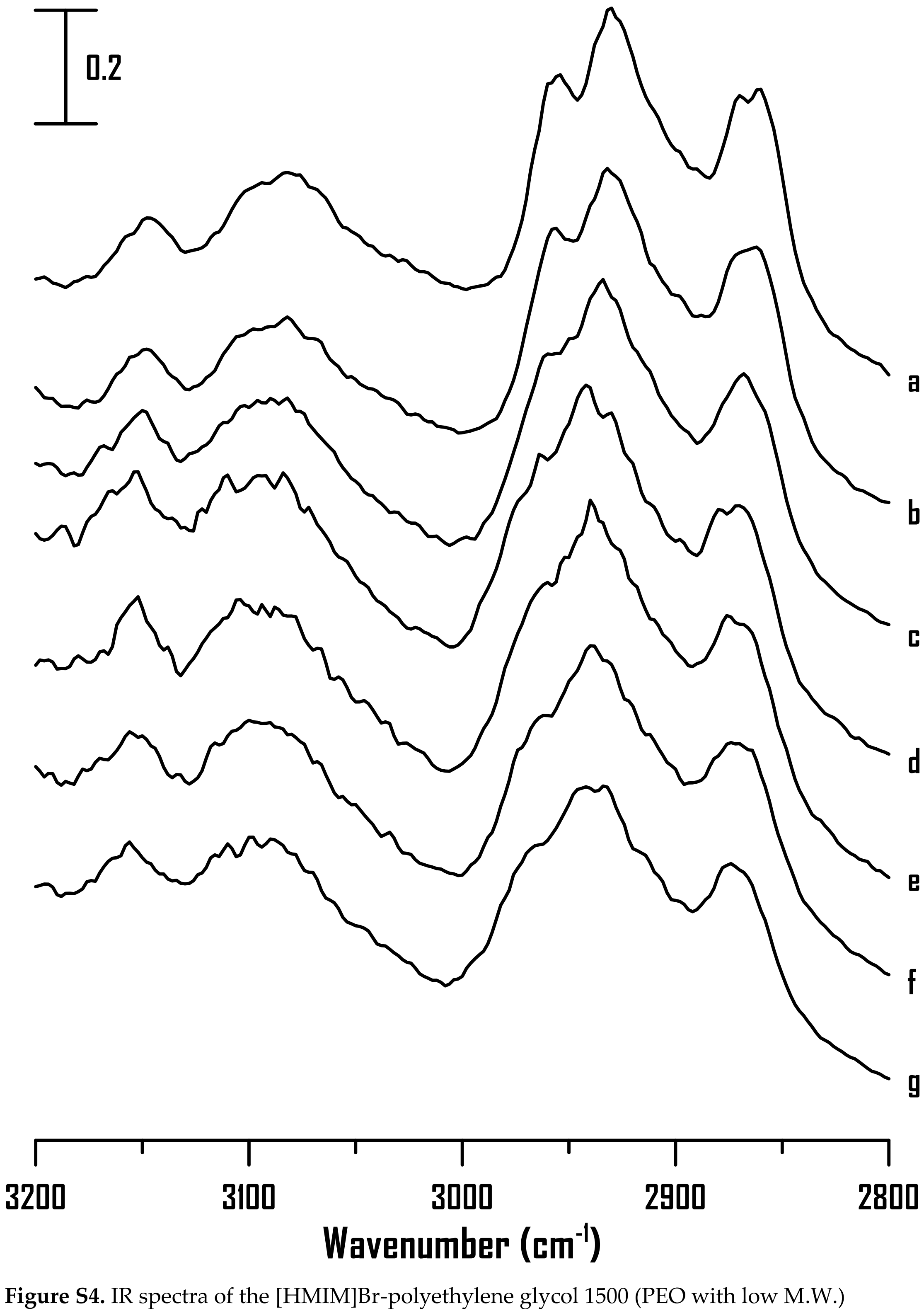

Figure S4. IR spectra of the [HMIM]Br-polyethylene glycol 1500 (PEO with low M.W.) mixture containing 80 wt% [HMIM]Br at (a) ambient pressure and (b) 0.4, (c) 0.7, (d) 1.1, (e) 1.5, (f) 1.8, and (g) 2.5 GPa.

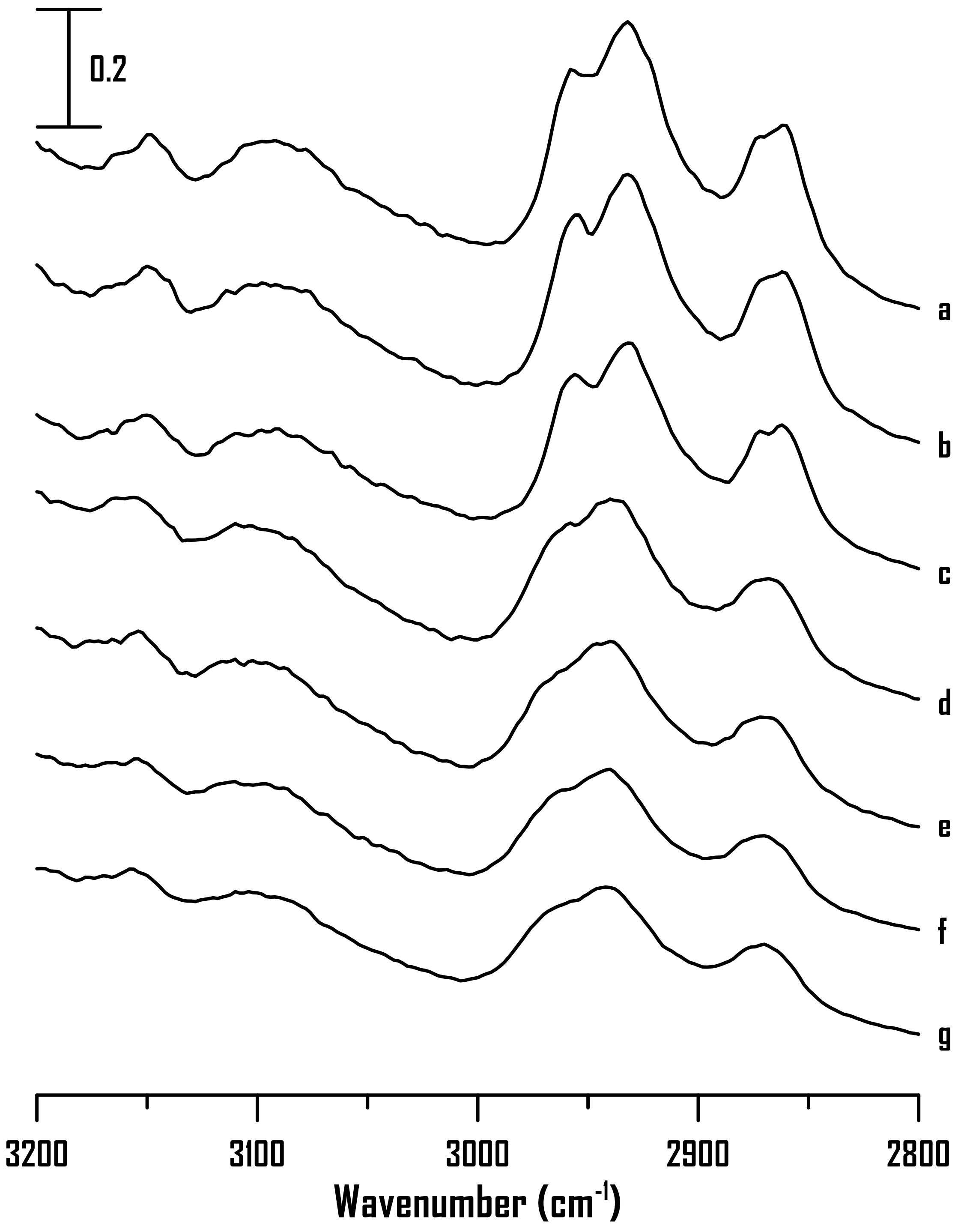

Figure S5. IR spectra of the [HMIM]Br-ethylene glycol mixture containing 80 wt% [HMIM]Br at (a) ambient pressure and (b) 0.4, (c) 0.7, (d) 1.1, (e) 1.5, (f) 1.8, and (g) 2.5 GPa.

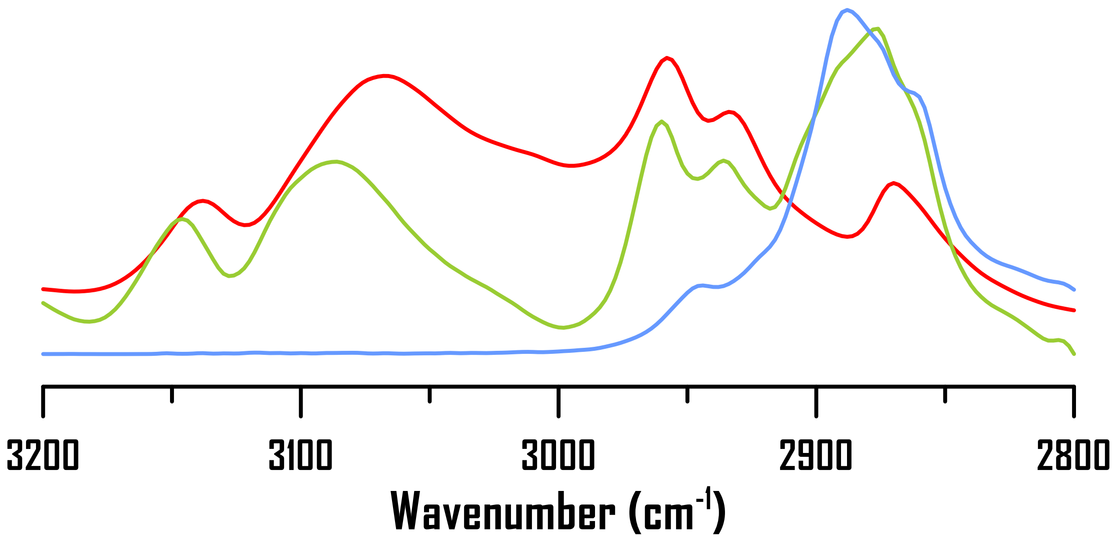

Figure S6 (overlaid spectra in Figure 1). Infrared (IR) spectra of (red) pure [BMIM]Br, (Martian green) mixture of PEO containing 75 wt% [BMIM]Br, and (baby blue) pure PEO at ambient pressure.

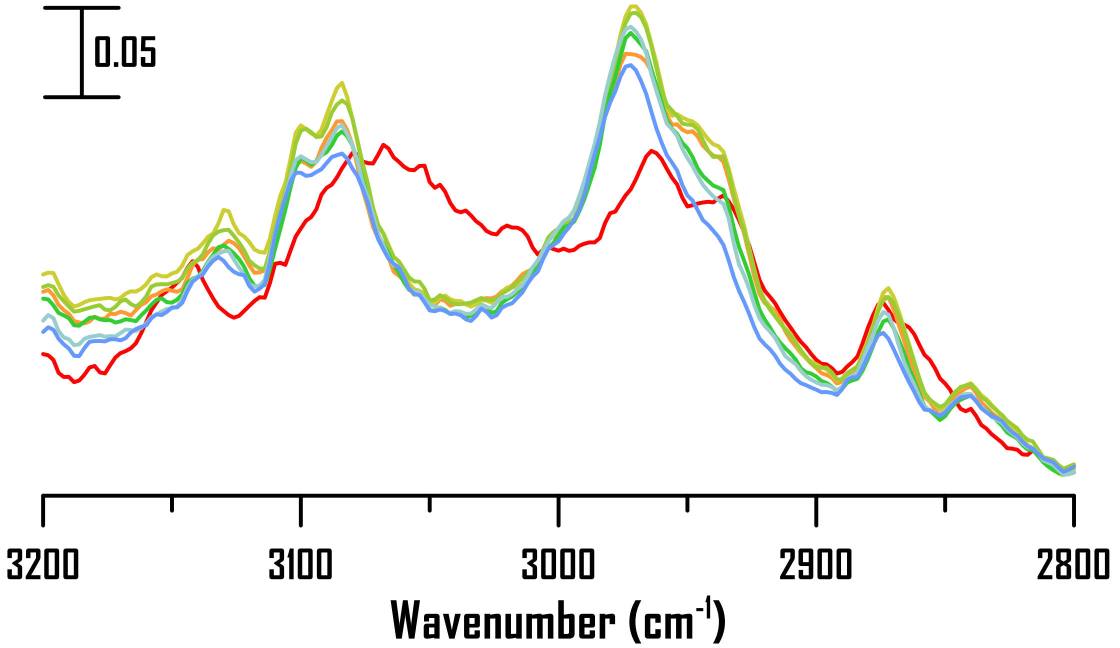

Figure S7 (overlaid spectra in Figure 3). IR spectra of pure [BMIM]Br at (red) ambient pressure and (light orange) 0.4, (banana yellow) 0.7, (Martian green) 1.1, (spring green) 1.5, (light Bluegreen) 1.8, and (baby blue) 2.5 GPa.

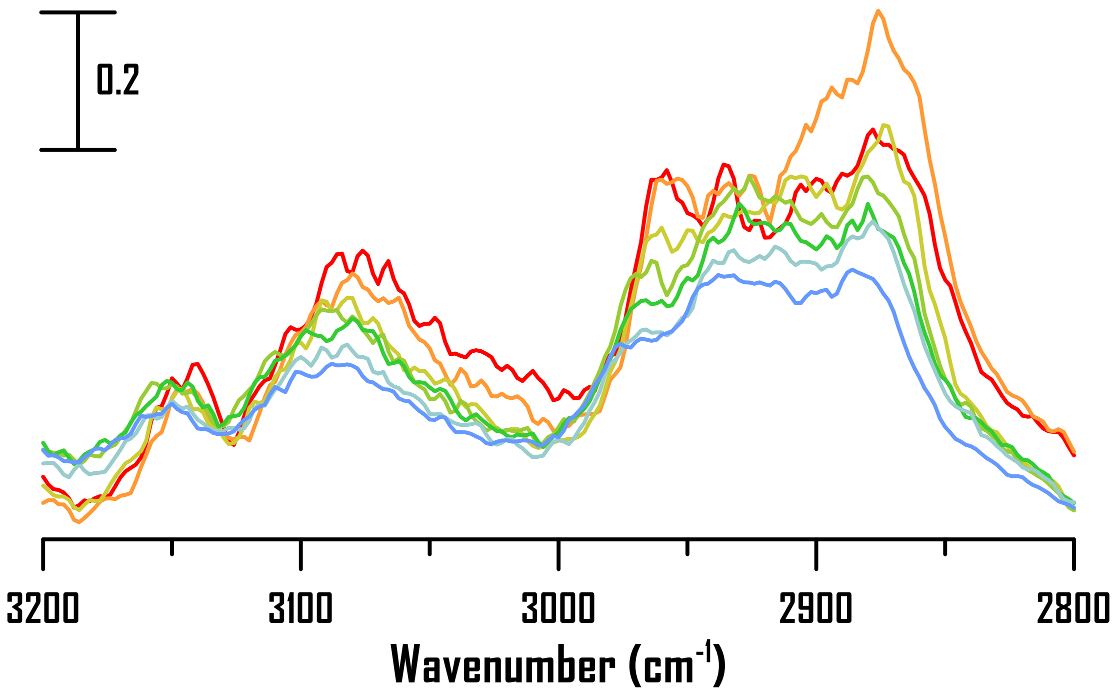

Figure S8 (overlaid spectra in Figure 4). IR spectra of the [BMIM]Br-PEO mixture containing 80 wt% of [BMIM]Br at (red) ambient pressure and (light orange) 0.4, (banana yellow) 0.7, (Martian green) 1.1, (spring green) 1.5, (light Bluegreen) 1.8, and (baby blue) 2.5 GPa.

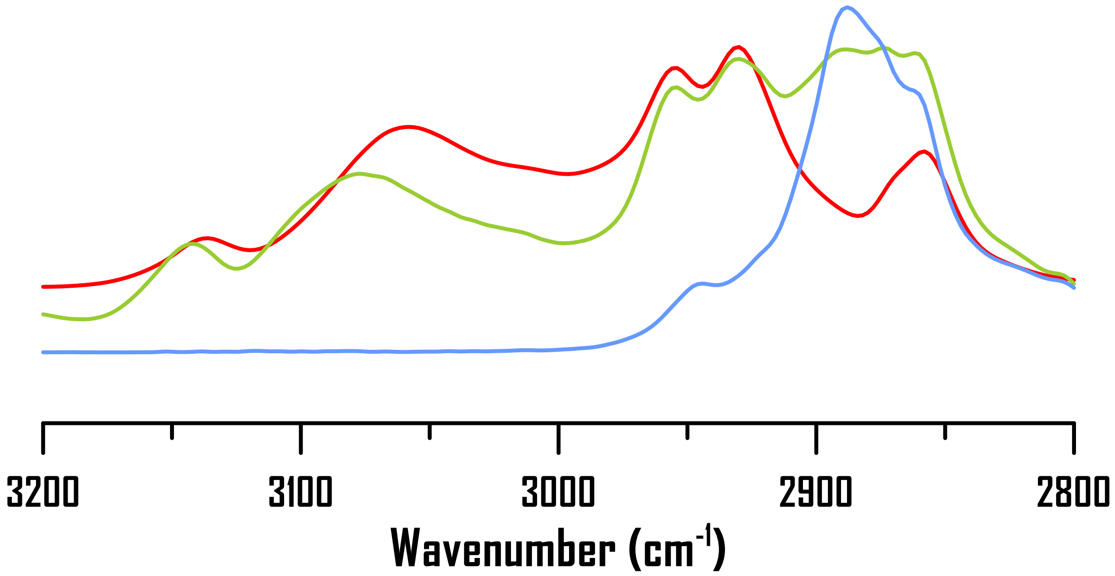

Figure S9 (overlaid spectra inFigure 6). IR spectra of (red) pure [HMIM]Br, (Martian green) mixture of PEO containing 75 wt% [HMIM]Br, and (baby blue) pure PEO at ambient pressure.

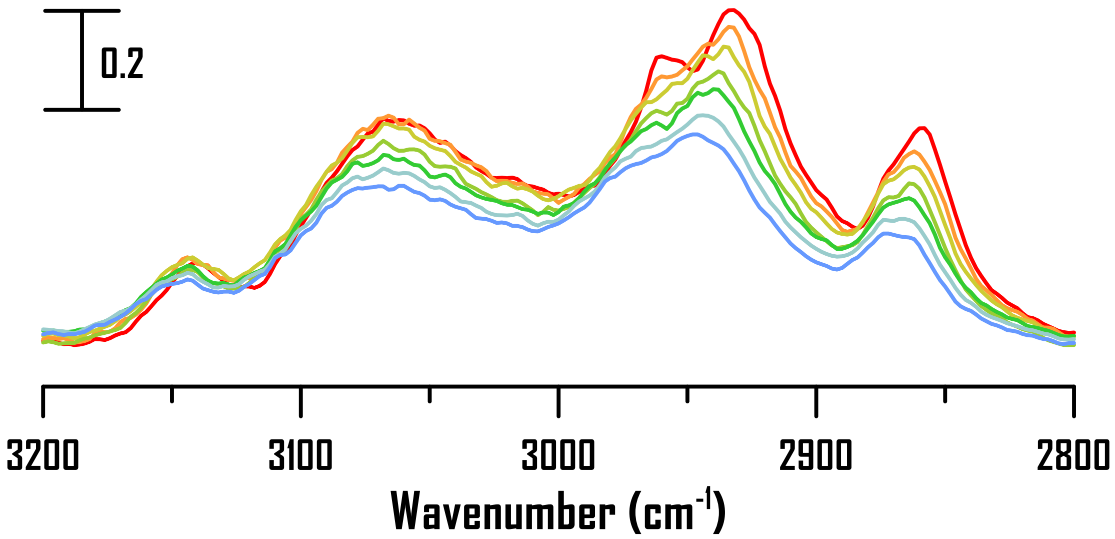

Figure S10 (overlaid spectra in Figure 8). IR spectra of pure [HMIM]Br at (red) ambient pressure and (light orange) 0.4, (banana yellow) 0.7, (Martian green) 1.1, (spring green) 1.5, (light Bluegreen) 1.8, and (baby blue) 2.5 GPa.

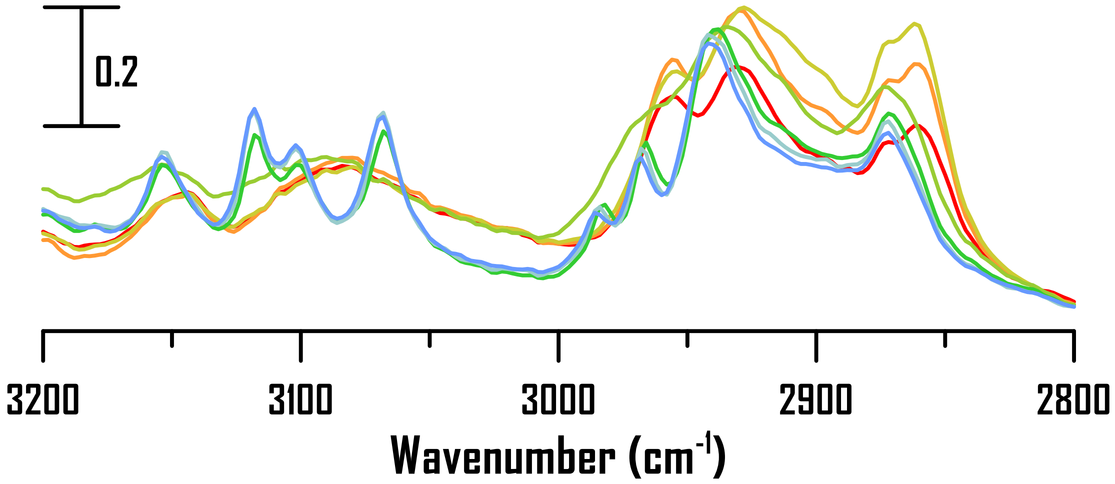

Figure S11 (overlaid spectra in Figure 9). IR spectra of the [HMIM]Br-PEO mixture containing 80 wt% [HMIM]Br at (red) ambient pressure and (light orange) 0.4, (banana yellow) 0.7, (Martian green) 1.1, (spring green) 1.5, (light Bluegreen) 1.8, and (baby blue) 2.5 GPa.
